# A collagen gel-coated, aligned nanofiber membrane for enhanced endothelial barrier function

**DOI:** 10.1038/s41598-019-51560-8

**Published:** 2019-10-17

**Authors:** Dohui Kim, Seongsu Eom, Sang Min Park, Hyeonjun Hong, Dong Sung Kim

**Affiliations:** 1 Department of Mechanical Engineering, Pohang University of Science and Technology (POSTECH), 77 Cheongam-ro, Pohang, Gyeongbuk 37673 South Korea; 2 Present Address: Department of Mechanical Engineering, Pusan National University, 2 Busandaehak-ro 63beon-gil, Busan, 46241 South Korea

**Keywords:** Biomedical engineering, Lab-on-a-chip

## Abstract

Herein, a collagen gel-coated and aligned nanofiber membrane named Col-ANM is developed, which remarkably improves endothelial barrier function by providing biochemical and topographical cues simultaneously. Col-ANM is fabricated by collagen gel coating process on an aligned polycaprolactone (PCL) nanofiber membrane, which is obtained by a simple electrospinning process adopting a parallel electrode collector. Human umbilical vein endothelial cells (HUVECs) cultured on Col-ANM exhibit remarkably enhanced endothelial barrier function with high expression levels of intercellular junction proteins of ZO-1 and VE-cadherin, a high TEER, and a cellular permeability compared with the artificial porous membranes in commercial cell culture well inserts. The enhanced endothelial barrier function is conjectured to be attributed to the synergistic effects of topographical and biochemical cues provided by the aligned PCL nanofibers and collagen gel in the Col-ANM, respectively. Finally, the reactive oxygen species is applied to the HUVEC monolayer formed on the Col-ANM to destroy the tight junctions between HUVECs. The destruction of the tight junctions is demonstrated by the decreased TEER value over time. Results indicate the potential of Col-ANM in modeling endothelial barrier dysfunction-related diseases.

## Introduction

The vascular endothelium, which is located in the innermost layers of blood vessels, plays a central role in the regulation of selective mass transport between blood vessels and surrounding tissues. The regulatory function of the endothelium, also called endothelial barrier function, maintains tissue homeostasis and protects tissues from infiltrating toxins^1,2^. If the disruption of endothelial barrier occurs, uncontrolled mass transport may bring various diseases including atherosclerosis, proteinuria, and Alzheimer’s disease^3–6^. Thus, understanding endothelial barrier function is essential to the elucidation of pathophysiological mechanisms or testing of drug candidates. The endothelial barrier function is affected by *in vivo* biophysical and biochemical microenvironment, such as chemical, mechanical, and topographical cues of the extracellular matrix (ECM); interaction between neighboring cells; shear stress by interstitial or blood flow; and mechanical strain^7–10^. In this regard, various microfabrication technologies for realizing endothelial barrier function on *in vitro* models have been developed with cell culture well inserts like Transwell, microfluidics chips, and 2D micro/nano-engineered substrates^10–13^.

Well inserts are highly reliable, low cost, and easy to use and have good accessibility through the standardization on commercial products. Commercial well inserts possess artificial porous membranes that enable the division of chambers into apical and basolateral sides, which constitute the tissue-tissue interfaces across the membranes. Well inserts which have free-standing and permeable porous membranes are thus suited for barrier function assays for measuring interfacial barrier integrity, transendothelial electrical resistance (TEER), and small-molecule permeability. Previous studies easily constructed endothelial barriers for various blood vessel-tissue interface models, including blood retinal barrier, blood-brain barrier, and pulmonary air-liquid interface^8,11,14^. However, commercial well inserts only provide limited insights into endothelial barrier function^15–18^ because the behavior of endothelial cells (ECs) on 2D flat porous membranes with limited permeability induced by artificially distributed pores are significantly different from the behavior of ECs on highly permeable and nanofibrous *in vivo* ECM membrane^19–21^.

Electrospinning is a simple and versatile way to fabricate a 3D nanofiber mat that emulates the nanofibrous native ECM structure. By modulating the chemical and topographical properties of an electrospun nanofiber membrane, the biochemical and/or biophysical cues can be implemented in an *in vitro* cell culture platform^22–24^. Various methods, including gel coating, surface modification, and blending hydrogel/polymer, have been developed for the incorporation of natural hydrogels, such as collagen and gelatin, into nanofiber membranes for the purpose of promoting EC proliferation and endothelial barrier formation through biochemical cues^23–27^. Electrospinning can control surface topography to facilitate the construction of endothelial barrier by modulating the diameter and alignment of nanofibers^21,28–31^. In uniaxially aligned nanofibers, the alignment of nanofibers promotes endothelial barrier integrity by inducing the aligned morphology of endothelium observed in *in vivo* microenvironments^21,24–28^. Owing to the unique advantages of the electrospun nanofiber membrane, many researchers have attempted to incorporate free-standing nanofiber membranes into well inserts to construct *in vitro* barrier models as substitutes for artificial porous membranes^27,32^. However, previous works were limited to the use of random nanofiber membranes in well inserts due to issues in the handling and integration of thin nanofiber membranes. This approach cannot induce the alignment of endothelium.

Here, we developed a cell culture well insert with a collagen gel-coated and aligned nanofiber membrane, which we named Col-ANM, for the construction of an effective endothelial barrier model. We fabricated an aligned polycarprolactone (PCL) nanofiber membrane, denoted by PCL-ANM, and integrated it on a custom-made 12 well insert wall in a free-standing configuration through a simple electrospinning process adopting a parallel electrode collector. We intended not only to align the ECs through the instructive topographical cue but also to define the apical and basolateral sides for developing an *in vitro* endothelial barrier model. Type I collagen gel was coated on the PCL-ANM for the fabrication of Col-ANM to promote endothelial barrier function by emulating the *in vivo* biochemical microenvironment. To validate the developed system, we cultured human umbilical vein endothelial cells (HUVECs) on Col-ANM and assessed barrier function through various ways, including evaluating intercellular junctions and the alignment of HUVECs based on immunostaining and measuring TEER and permeability. Col-ANM showed better performance than the artificial porous membranes in developing the endothelial barrier model. The destruction of the tight junctions between HUVECs over time in response to reactive oxygen species (ROS) treatment suggests the potential of Col-ANM in disease modeling.

## Results and Discussion

### Fabrication of Col-ANM well insert

**Figure 1:**
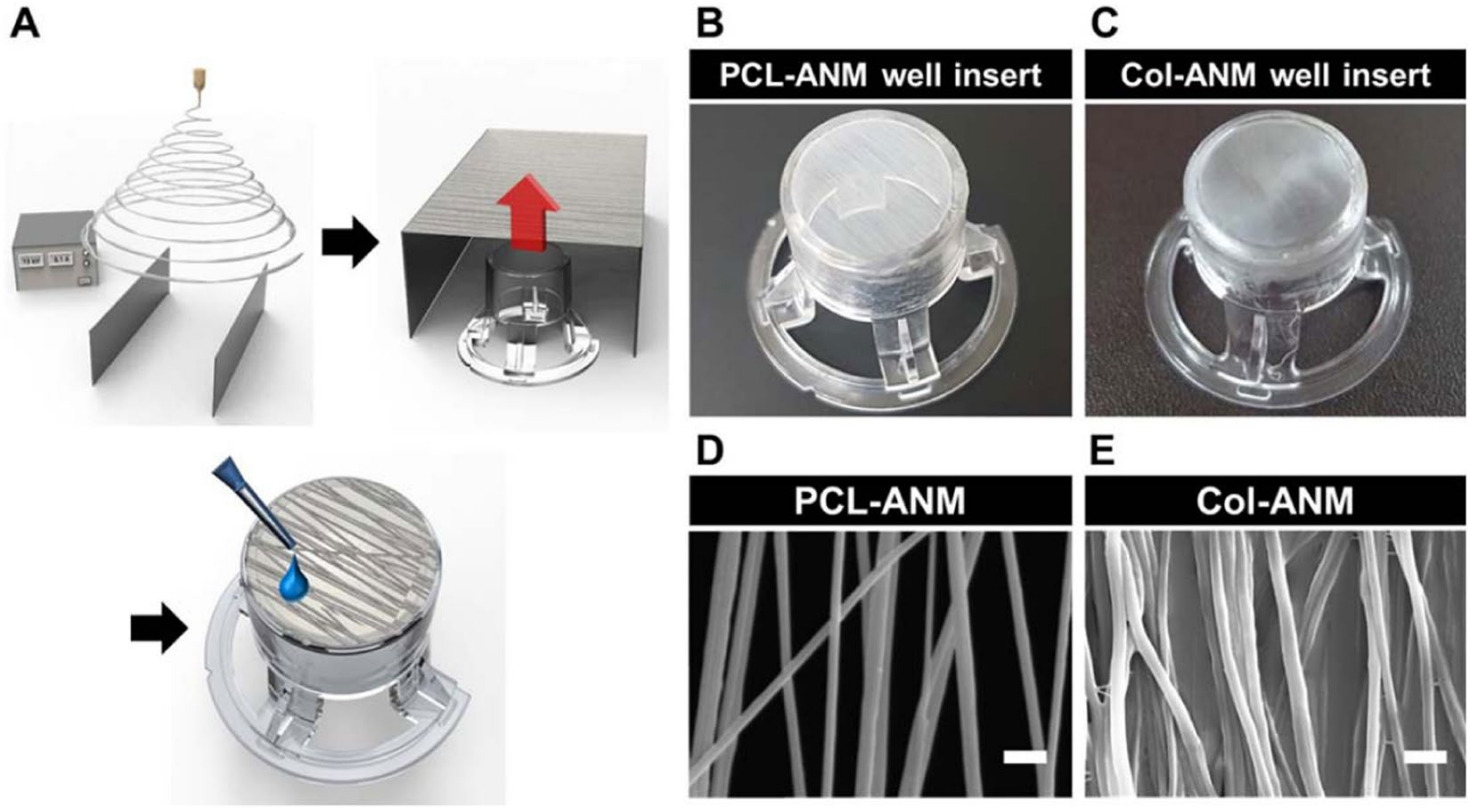
Fabrication process of Col-ANM well insert. (**A**) Schematic diagram of the experimental setup and the fabrication of Col-ANM well insert. Photographs of (**B**) PCL-ANM well insert and (**C**) Col-ANM well insert. SEM images of (**D**) PCL-ANM and (**E**) Col-ANM. Scale bars are 2 μm.

Figure 1A shows the schematic of the fabrication process of a Col-ANM on a cell culture well insert. In the first step, by employing a collector with two parallel electrodes, we successfully fabricated a PCL-ANM between the two parallel electrodes. The electric field generated by the parallel electrode collector resulted in the alignment of the as-spun nanofibers perpendicular to the electrode owing to the electrostatic forces on the fibers^33^. PCL-ANM was carefully transferred to the bottom opening of the custom-made 12-well insert wall with no membrane. Figure 1B shows the free-standing feature of the PCL-ANM, which is necessary not only to the construction of the blood vessel-tissue interface but also to the evaluation of the interfacial barrier integrity, TEER value, and permeability. In the last step, the basolateral side of the PCL-ANM was coated with collagen gel, a major component of the ECM, to enrich the nanofiber membrane with biochemical components for the production of the Col-ANM well insert (Fig. 1C). The collagen gel coating process was devised to retain nanofiber topography on the apical side of the membrane and minimize the amount of collagen gel. During the collagen gelation process, the addition of 1 M NaOH and 10 × Dulbecco’s modified eagle medium enabled the sufficient supply of ions and neutralization to induce the assembly of collagen molecules to form collagen nanofibrils and entanglement of the collagen nanofibrils. Moreover, the increase in temperature further induced the entanglement of collagen nanofibrils to generate a rapid sol-gel transition. The SEM images show the nanofibrous structures of the fabricated PCL-ANM (Fig. 1D) and Col-ANM (Fig. 1E) on their apical sides.

### Characterization of Col-ANM in comparison with commercial porous membranes

**Figure 2:**
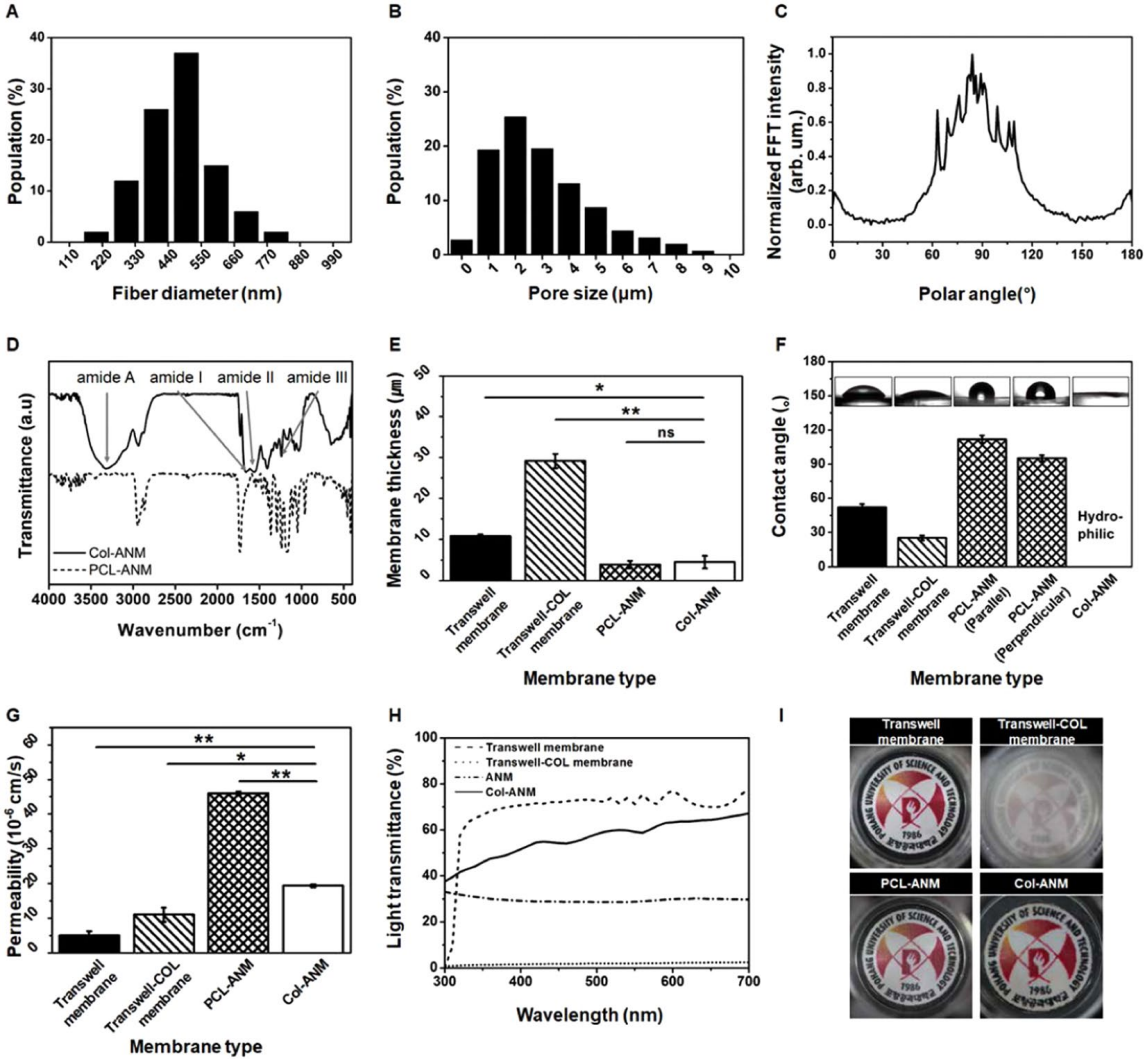
Characterization of Col-ANM. (**A–D**) Physical properties of nanofibers in PCL-ANM and Col-ANM. (**A**) Fiber diameter distribution of the nanofibers. (**B**) Pore size distribution of the PCL-ANM. (**C**) Normalized fast Fourier transform intensity representing the aligned structure of nanofibers. (**D**) FTIR spectra of the PCL-ANM (dashed line) and Col-ANM (solid line). (**E**–**I**) Comparison of physical properties of Col-ANM and PCL-ANM with respect to Transwell membrane and Transwell-COL membrane. (**E**) Membrane thickness of the four different membranes. (**F**) Contact angle of the four different membranes. (**G**) Membrane permeability of the four different membranes. (**H**) Light transmittance of the four different membranes. (**I**) Photographs showing transparency of the four different membranes.

Figure 2 shows the various physical properties of the four different membranes, which included two different types of nanofiber membranes (PCL-ANM and Col-ANM) and two different artificial porous membranes (Transwell and Transwell-COL membranes). The fiber diameter of the PCL-ANM ranged from 200 nm to 800 nm, with an average diameter of 477.00 ± 120.48 nm, as plotted in Fig. 2A. This result shows that the nanofibers of the PCL-ANM had a comparable dimension with the collagen fibrils of the native ECM (50–500 nm). The analysis on the SEM images revealed that the PCL-ANM had an average porosity of 59.64 ± 1.75% and an average pore size of 3.58 ± 1.95 μm, whereas the Transwell membrane possessed an average porosity of 1.59 ± 0.12% and the predefined pore size of 0.4 μm (Supplementary Fig. S1). The pore size distribution of PCL-ANM was presented in Fig. 2B. Further, the possibility to tune the porosity and the pore size of PCL-ANM was also demonstrated (Supplementary Fig. S2). The result of FFT, indicated in Fig. 2C, demonstrated that the PCL-ANM had a uniaxially aligned nanofiber structure, which was expected to induce cell alignment. The existence of collagen gel on the surface of the Col-ANM after collagen gel coating on the PCL-ANM was confirmed by examining both the PCL-ANM and Col-ANM by FTIR spectra. The infrared spectra for both the PCL-ANM and Col-ANM in Fig. 2D showed the characteristic bands of PCL such as carbonyl stretching (1,729 cm^−1^), asymmetric CH_2_ stretching (2,952 cm^−1^), and symmetric CH_2_ stretching (2,868 cm^−1^). In addition, as indicated by arrows in Fig. 2D, the Col-ANM possessed additional bands for amide A (3,333 cm^−1^), amide I (1,658 cm^−1^), amide II (1,548 cm^−1^), and amide III (1,238 cm^−1^), which are typical peaks of collagen. These results validated that the collagen gel was successfully coated on the surface of the PCL-ANM in the Col-ANM. For the additional peaks other than the characteristic bands of PCL and collagen gel observed in the FTIR spectra of Col-ANM, we conjectured that the PCL nanofibers were physically embedded in the collagen gel like a composite material. It was also expected that the Col-ANM would possess physically entangled fibrillar structures because the collagen gelation process induces the assembly of collagen molecules to form collagen nanofibrils and further entanglement of collagen nanofibrils. Moreover, considering that the collagen gels were firmly attached after 4 days of cell culture, we could determine that the adhesion between the collagen gel and the PCL-ANM is sufficiently strong.

The thickness of PCL-ANM was 3.90 ± 0.92 μm and that of Col-ANM was 4.50 ± 1.55 μm (Fig. 2E). This result suggested that the present collagen gel coating process did not significantly increase the thickness of PCL-ANM. The Transwell and Transwell-COL membranes were 10.78 ± 0.43 and 29.15 ± 1.75 μm thick, respectively, which were more than 2 times thicker than the Col-ANM. Given that the thickness of the *in vivo* endothelial basement membrane is about 50–100 nm, the Col-ANM can provide a more *in vivo* comparable thickness to the cells than the two artificial porous membranes.

Because the membranes with hydrophilicity are favorable for cell adhesion^34^, we measured contact angles to evaluate the hydrophilicity of the four different membranes. Figure 2F exhibits the superior hydrophilicity of the Col-ANM compared with the PCL-ANM and two artificial porous membranes of Transwell and Transwell-COL. The contact angles of the Transwell membrane, Transwell-COL membrane, PCL-ANM (Parallel) and PCL-ANM (Perpendicular) were measured to be 52.13 ± 2.74°, 25.23 ± 2.2°, 111.97 ± 3.36°, and 95.27 ± 2.57°, respectively. This result showed that the Col-ANM would provide a cell favorable environment that affects cell adhesion, survival, growth and differentiation^35,36^.

Exchange of comparatively large molecules, such as growth factors and cytokines, is essential for cellular interactions which greatly influence on diverse physiological and pathophysiological processes in blood vessel-tissue barrier. The macromolecules permeate through the basement membrane, which is found beneath the endothelium, by concentration gradient-driven diffusion. Given that human proteins are known to have a size of few tenths of kDa, we analyzed the membrane permeability (*P*_*m*_) using 40 kDa FITC-dextran as a representative macromolecule. By measuring diffusion of dextran molecules across the membrane, which was realized by an externally imposed concentration gradient in the apical chamber, we could compare the *P*_*m*_ of the four different membranes. Figure 2D showed that the *P*_*m*_ of PCL-ANM and Col-ANM (7.27 ± 0.58 × 10^−5^ cm/s and 1.94 ± 0.04 × 10^−5^ cm/s, respectively) were higher compared to Transwell and Transwell-COL membranes (4.99 ± 1.30 × 10^−6^ cm/s and 11.01 ± 2.02 × 10^−6^ cm/s, respectively). In the human body, the *P*_*m*_ of *in vivo* vascular basement membranes varies significantly with the location (i.e., 1.55 × 10^−5^ cm/s for 70 kDa dextran in the glomerular basement membrane^37^, and 3.5 × 10^−6^ cm/s for human serum protein in Bruch’s membrane^24^). By tuning the concentration (3.0 mg/ml) and amount (50 μl) of the collagen gel, we could realize the *P*_*m*_ of the Col-ANM to match with the order of *in vivo* vascular basement membrane. We expected that by changing the amount or concentration of the collagen gel, we could tune the permeability of the Col-ANM according to the target vascular basement membrane. Therefore, the Col-ANM can further realize the enhanced cellular interactions when the Col-ANM is employed in the development of *in vitro* models instead of artificial porous membranes.

The cells were examined with fluorescence microscopy at a suitable light transmittance. As shown in Fig. 2H,I, the Col-ANM and Transwell membrane provided more proper light transmittance compared with Transwell-COL and PCL-ANM. Although the light transmittance of the PCL-ANM was below 40% at 500 nm wavelength, the light transmittance of the Col-ANM was improved up to almost 60%. Increase in the light transmittance of the Col-ANM may be attributed to the filling of the air pockets within the nanofibers of PCL-ANM by the collagen gel. The smaller difference in refractive indices between PCL (1.437) and collagen gel (1.333) in the Col-ANM than that between PCL and air (1.0) in the PCL-ANM induced lesser light scattering, which resulted in the better light transmittance of Col-ANM as compared with that of PCL-ANM.

Because we developed the Col-ANM on a cell culture well insert, it could not reflect the fluid flow environment found in *in vivo*, which may negatively affect the endothelial barrier function. As a future work, by integrating the Col-ANM with the microfluidic devices, a more enhanced endothelial barrier function could be realized^9^. Moreover, further enhancement of the blood-tissue barrier could be expected through the co-culture with other tissue cells, such as astrocyte and podocyte, based on the active cellular interactions across the highly permeable Col-ANM^8,11^

### Formation of intercellular junction proteins and alignment of cells

**Figure 3:**
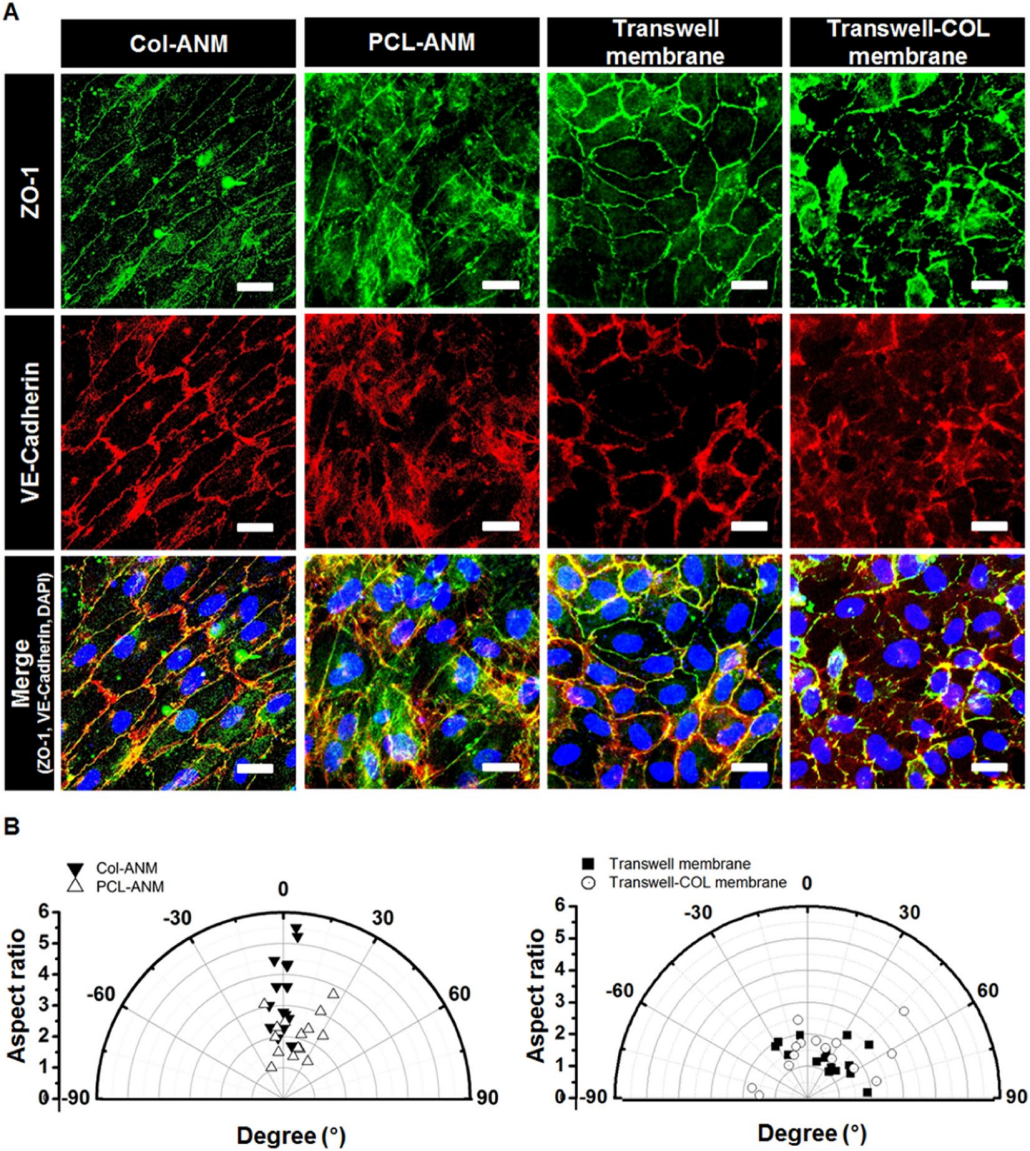
Characterization of intercellular junction proteins and cell morphology of the HUVECs monolayer. (**A**) Immunofluorescence images of HUVECs on the four different membranes. Adherens junctions and tight junctions were stained with VE-cadherin (red) and ZO-1 (green), respectively, and nuclei were stained with DAPI (blue). Scale bars are 20 μm. (**B**) Aspect ratio and orientation angle of HUVECs cultured on the four different membranes.

Endothelial junctions composed of tight junction and adherens junction generate adhesive contacts between adjacent cells. Tight junction is known to regulate the paracellular pathway for ion and solute transport between cells controlling the permeability of endothelium, and adherens junction plays an important role in initiating, mediating, and maintaining endothelial barrier integrity^38–41^. Immunofluorescence staining on ZO-1 and VE-cadherin revealed the presence of tight junction proteins and adherens junction proteins of the HUVECs, respectively. Given that ZO-1 only forms in the edge of cell-cell joints and VE-cadherin tends to move to the edge of a cellular surface from the cytoskeleton as the intercellular junction matures, the uniform and continuous signals on the edge of cell-cell joints verify the abundant junction proteins and strong barrier integrity of an endothelial monolayer^42,43^. The results shown in Fig. 3A demonstrated that the formation of ZO-1 and VE-cadherin in the HUVECs on the Col-ANM was more uniform than those on the PCL-ANM and the other two artificial porous membranes (Transwell and Transwell-COL membranes). After comparing PCL-ANM and Col-ANM in terms of junction protein expression, we confirmed that the biochemical components provided by collagen gel coating positively influenced the formation of the intercellular junction. Conclusively, the endothelial barrier integrity of the HUVECs on the Col-ANM was more enhanced than that on the other three membranes of PCL-ANM and artificial porous membranes.

Endothelial cells in *in vivo* are elongated and aligned along the blood vessels due to the shear stress of blood flow, and cellular alignment is associated with intercellular junctions and the functions of ECs^44,45^. In this study, the elongation and alignment of HUVECs were recapitulated in *in vitro* through the topographical cue provided by the aligned nanofibers of PCL-ANM and Col-ANM used to emulate *in vivo* ECs morphology in blood vessels. After characterizing the aspect ratio and orientation angle of the cells, we found that the HUVECs tended to be highly aligned on the Col-ANM whereas those on the Transwell and Transwell-COL membranes exhibited randomly distributed orientation angles with low aspect ratios (Fig. 3B). In addition, though the HUVECs on the PCL-ANM also showed some aligned morphology, it was not clear, and also the aspect ratios were not large enough because the PCL-ANM does not possess biochemical components to enhance cell adhesion on the surface^23^. These results showed that the HUVECs formed a highly aligned and elongated morphology on Col-ANM and emulated the morphology of the *in vivo* vascular endothelium owing to the synergistic effect of topographical cue and biochemical components provided by the aligned nanofibers and the collagen gel, respectively.

### Evaluation of TEER value and cellular permeability

**Figure 4:**
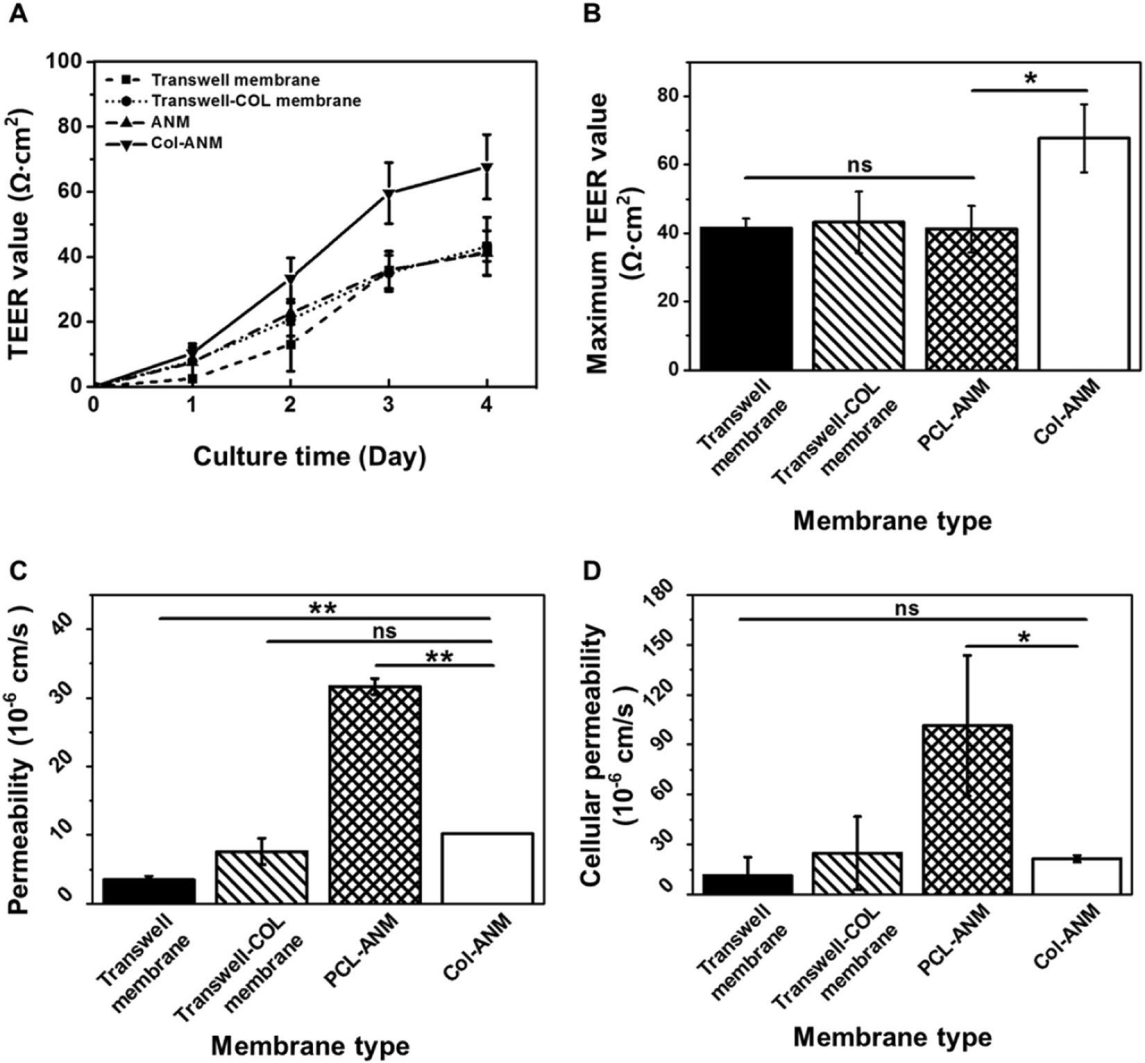
Evaluation of the endothelial barrier integrity of HUVECs monolayer on ANM compared with artificial porous membranes. (**A**) Change of TEER values of the HUVECs monolayer on the four different membrane during four days of culture. (**B**) Maximum TEER values of the HUVECs monolayers on the four different membranes. (**C**) Apparent permeability of the four different membranes with HUVECs monolayers. (**D**) Cellular permeability of the HUVECs monolayers on the four membranes. The values shown are means ± SD (n = 3). NS represents that the values have no statistical differences.

Although a high TEER value comparable to the *in vivo* condition is necessary to the development of a reliable *in vitro* test model, the TEER values obtained only with ECs in previous works showed limited levels^15–18^. In this regard, constructing an *in vitro* platform with a high TEER value remains challenging^45^. Furthermore, as the TEER value should be measured between both sides of a membrane, the free-standing feature of the model is inevitably required^46^. The integration of Col-ANM into a cell culture well insert wall afforded the free-standing feature of the nanofiber membrane and thereby allowed the measurement of the TEER values of the HUVECs. The cell culture well inserts with four different membranes were tested for the evaluation of the TEER values. As shown in Fig. 4A,B, the TEER values of the HUVEC monolayer on the Col-ANM (67.70 ± 9.94 Ω·cm^2^) showed a remarkably higher TEER value than those of the HUVEC monolayer on Transwell and Transwell-COL (41.44 ± 2.82 and 43.18 ± 8.96 Ω·cm^2^, respectively). This improvement verified the enhanced endothelial barrier function on Col-ANM compared with commercial porous membranes owing to the synergistic effect of the biochemical and topographical cues on the cellular functions. Considering that the maximum TEER value of the PCL-ANM (41.19 ± 6.79 Ω·cm^2^) has no significant difference compared to the Transwell membrane, we could conclude that the nanofibrous topographical cue showed only marginal effect on the enhancement of endothelial barrier function. For the case of biochemical cue, the similar TEER values between Transwell membrane and Transwell-COL membrane indicated the insignificant effect of the biochemical cue on the endothelial barrier function. Interestingly, the provision of both the topographical and biochemical cues through the Col-ANM allowed the great increase in the TEER value, which demonstrated the synergistic effect of the topographical and biochemical cues on the endothelial barrier function. We further compared the TEER values of the HUVECs monolayer on the Col-ANM and a collagen gel-coated and random nanofiber membrane (Col-RNM) which represented the case of the different environment with different type of nanofibrous topography with the same biochemical cue. The improved TEER value on the Col-ANM compared with PCL-ANM indicated the importance of the aligned topographical cue to construct an improved endothelial barrier (Supplementary Fig. S3). Therefore, the results indicated the importance of synergistic effect on enhanced endothelial barrier function, which can be achieved by the topographical and biochemical cues of Col-ANM.

Measuring the solute permeability of tracer molecules provides an evaluation tool for the maturation of a cellular monolayer. Permeability is one of the important parameters for developing *in vitro* drug test models and estimating drug transport *in vivo* at the early stage of drug discovery^47^. In a drug test, a weak barrier function may result in an imprecise prediction of drug transport in the human body. A low cellular permeability coefficient (*P*_*c*_) represents the strong integrity of endothelial monolayer to be used for model development. For the characterization of the cellular permeability coefficient, the apparent permeability coefficients (*P*_*a*_) of the four different membranes were measured, which were found to be 10.22 ± 0.53 × 10^−6^, 31.20 ± 1.16 × 10^−6^, 3.47 ± 0.55 × 10^−6^, and 7.63 ± 1.91 × 10^−6^ cm/s for Col-ANM, PCL-ANM, Transwell, and Transwell-COL membranes, respectively (Fig. 4C). By excluding the membrane permeability coefficients (*P*_*m*_), the *P*_*c*_ of the HUVEC monolayer cultured on the membranes was obtained based on Eq. (3), as plotted in Fig. 4D. The *P*_*c*_ of the Col-ANM had a value of 21.56 ± 2.2 × 10^−6^ cm/s, which was comparable with the *P*_*c*_ values of the Transwell and Transwell-COL membranes (11.39 ± 11.13 × 10^−6^, and 24.85 ± 21.85 × 10^−6^ cm/s, respectively). Owing to the remarkably high *P*_*m*_ and rough surface of PCL-ANM, the *P*_*c*_ of PCL-ANM showed the highest value of 101.40 ± 42.13 × 10^−6^ cm/s. However, although Col-ANM had a higher *P*_*m*_ and the rough surface compared with the low *P*_*m*_ and the flat surface of the commercial porous membranes, the topographical and biochemical cues of Col-ANM promoted the maturation of the HUVEC monolayer to an extent comparable to that observed on the commercial membranes.

### Loss of endothelial barrier integrity by ROS treatment

**Figure 5:**
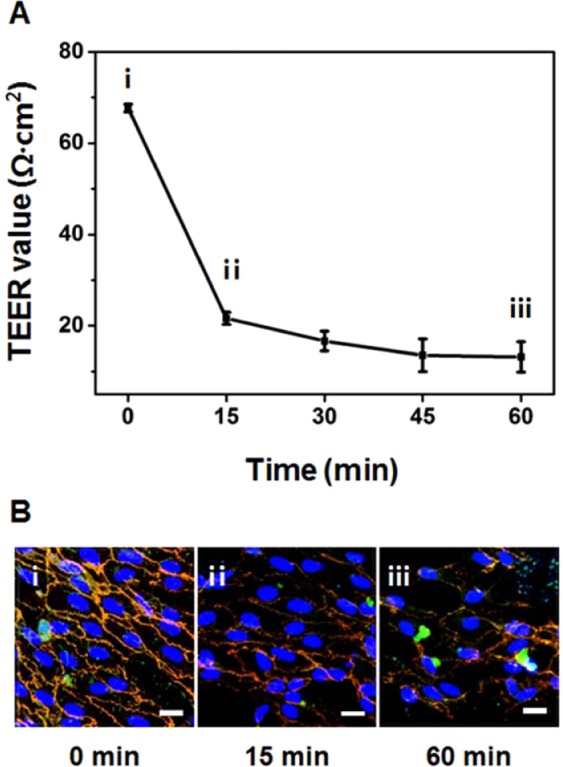
Effect of ROS treatment on TEER values and intercellular junctions of HUVECs monolayer on the Col-ANM. (**A**) Change in TEER values over time after ROS treatment. (**B**) Immunofluorescence images of HUVECs after ROS treatment: before ROS treatment (B-i), 15 min after ROS treatment (B-ii), and 60 min after ROS treatment (B-iii). Adherens junctions and tight junctions were stained with VE-cadherin (red) and ZO-1 (green), respectively, and nuclei were stained with DAPI (blue). Scale bars are 20 μm.

ROS weakens cell-cell junctions in native endothelium by mediating the Rac-induced signaling pathway^48^. ROS treatment is related to the pathophysiology of endothelial damage and inflammation, so we modeled the leakage of the endothelial barrier of the HUVECs cultured on Col-ANM by treating ROS. On day 5, when the integrity of tight junctions and adherens junctions was maximized, we induced endothelium dysfunction through ROS treatment. The TEER value rapidly decreased from 67.70 Ω·cm^2^ to 21.65 Ω·cm^2^ within 15 min after the ROS treatment. Further decrease in the TEER value was observed down to 13.19 Ω·cm^2^ after 1 h of treatment, as shown in Fig. 5A. The immunofluorescence stains of the intercellular junction proteins validated the disruption of cell-cell junctions caused by the ROS treatment, as shown in Fig. 5B. Before the ROS treatment, the orange intercellular junctions were detected by co-localizing the ZO-1 (green) and VE-cadherin (red), as shown in Fig. 5B(i). However, after 1 h of ROS treatment, loss of intercellular junctional proteins because of ROS was observed (Fig. 5B(ii,iii)). This result demonstrated the potential usability of Col-ANM as one of the test platforms to model the endothelium dysfunction. This dysfunctional endothelium model can be further improved for the development of *in vitro* blood-tissue barrier models for drug development.

## Conclusion

We developed a Col-ANM cell culture well insert to improve endothelial barrier function. The integration of Col-ANM with the well insert in a free-standing configuration enabled not only the effective construction of an *in vitro* blood-tissue interface but also the evaluation of interfacial barrier integrity. Owing to the synergistic effects of the topographical and biochemical cues of Col-ANM, the HUVECs exhibited enhanced endothelial barrier functions in terms of intercellular junction proteins, cell morphology, TEER value, and cellular permeability. Defective endothelium functions were simulated on Col-ANM as an application example of the endothelial barrier disease modeling. Given that Col-ANM provided a relevant tool for improving the endothelial barrier, it can be widely used for the development of reliable *in vitro* blood-tissue barrier models in biomedical and pharmaceutical fields.

## Materials and Methods

### Materials

PCL (average *M*_*n*_ = 80,000), chloroform, and methanol were purchased from Sigma-Aldrich (USA). Rat-tail type I collagen (3.59 mg/ml in acetic acid), 12 well Transwell insert (0.4 µm pore size), and 12 well Transwell-COL insert (0.4 µm pore size) were obtained from Corning (USA). VE-cadherin (D87F2) XP was supplied by Cell Signaling Technology (USA). The materials of Transwell membrane and Transwell-COL membrane were polyethylene terephthalate (PET) and polytetrafluoroethylene (PTFE), respectively. The collagen treatment on Transwell-COL generated a nano-fibrillar structure instead of a molecular-scale coated planar structure and did not alter the porous structure of the Transwell membrane. ZO-1 monoclonal antibody (ZO1-1A12) and DAPI (4′,6-Diamidino-2-Phenylindole, Dihydrochloride) were purchased from Thermo Fisher Scientific (USA). Hydrogen peroxide (H_2_O_2_, 30.0–35.5%) was obtained from Samchun Chemical (South Korea).

### Fabrication of Col-ANM well insert

The PCL-ANM was fabricated by a simple electrospinning process. For the preparation of a PCL solution, PCL pellets were dissolved in chloroform/methanol (3:1 vol/vol) solvent with 7.5% concentration. The PCL solution was loaded into a glass syringe, which was connected to a 23-gauge metal nozzle (NanoNC, South Korea), and fed at a constant flow rate of 0.7 ml/h by a syringe pump (KDS200, KD Scientific, USA). A parallel electrode collector consisting of two metal plates separated 30 mm apart was applied for the preparation of an aligned nanofiber membrane and was set 20 cm apart from the nozzle tip. As-electrospun PCL nanofibers were deposited between the two metal plates in an aligned manner after a high voltage (19 kV) was applied between the nozzle tip and collector with a voltage supplier (HV30, NanoNC). The deposited PCL-ANM was then detoxified under vacuum for 12 h. Then, the PCL-ANM was carefully transferred to a custom-made 12-well insert wall that did not possess a membrane.

For the collagen gel coating process, a neutralized collagen solution was prepared by mixing rat tail type I collagen, 1 M NaOH, and 10 × Dulbecco’s modified eagle medium (Sigma-Aldrich). The final concentration of the collagen solution was set to 3.0 mg/ml. Exactly 50 µl of the collagen solution was applied to the basolateral side of the PCL-ANM to cover the membrane surface uniformly. After incubation at 37 °C at 0.5% CO_2_ for 30 min, the collagen solution formed collagen gel on the PCL-ANM, resulting in a Col-ANM on the well insert.

### Characterization of Col-ANM

Before evaluating the morphology and thickness of the Col-ANM, the membrane was washed with deionized (DI) water 3 times after collagen gelation and dried at room temperature for 1 day. The morphology of the PCL-ANM and Col-ANM was examined by field-emission scanning electron microscopy (FE-SEM, SU6600, Hitachi, Japan). The fiber diameter of the electrospun nanofibers was measured with ImageJ analysis software (NIH, USA) based on the SEM images of the PCL-ANM. The porosity of membrane was calculated by dividing the area of the membrane excluding the pores by the total membrane area. The pores were randomly chosen in the SEM image and the pore diameter (*D*) was determined by using $D={[(4\times {\rm{A}})/{\rm{\pi }}]}^{1/2}$, where *A* is the pore area, assuming a circular pore. Though the pore shape is far from the circular shape for the case of nanofiber membranes, but it can provide a practical way to characterize the pore size of the PCL-ANM. The alignment of nanofibers was analyzed by applying fast Fourier transform (FFT) on the binarized SEM image of the PCL-ANM. The presence of a peak indicating the alignment of the nanofibers was determined by plotting the radial sum of the normalized FFT intensity with respect to polar angle. Col-ANM, PCL-ANM, and two commercial porous membranes, Transwell membrane and Transwell-COL membrane, were compared in terms of membrane thickness, permeability, and light transmittance. A Fourier transform infrared spectrophotometer (FTIR, Vertex 70, Bruker, Germany) was utilized to analyze the chemical structures of the PCL-ANM and the Col-ANM. The samples were rinsed with DI water, dehydrated in a graded ethanol series (30, 50, 70, 90, and 100%) and lyophilized with a freeze-dryer. The FTIR analysis was conducted with a resolution of 4 cm^−1^ in a range from 4,000 to 400 cm^−1^. For the measurement of the thickness of the sample, the membrane was fixed in a mixture of polydimethylsiloxane (PDMS) monomer and a curing agent (10:1, weight ratio) and baked at 55 °C for 24 h. The cross-sectional image of the membrane fixed inside the PDMS block was captured by optical microscopy, and the membrane thickness was measured by ImageJ analysis software. The contact angles were measured by the sessile drop method. To evaluate the contact angle, a water droplet (10 μl) was gently deposited on the sample surface using an automated contact angle measurement system (SmartDrop, Femtofab, South Korea). For the PCL-ANM, the contact angles were measured in both perpendicular and parallel directions to the alignment of nanofibers. Permeability was evaluated by measuring the diffusion of 40 kDa FITC-dextran tracers across the membrane. The four types of well inserts with different membranes were placed in a 12-well plate. Then, 1.5 ml of phosphate buffered saline (PBS) was pour onto the basolateral side of the well insert, and 0.5 ml of 200 µg/ml FITC-dextran solution was added to the apical side of the well insert. After 1 h at room temperature, 100 µl of the supernatant of the sample solution was collected from the basolateral side and placed in a 96-well plate. A fluorescence image of the 96-well chamber was captured with a phase-contrast inverted fluorescence microscope (Eclipse TS100, Nikon, Japan) and analyzed with a custom-coded MATLAB program. The membrane permeability coefficient was determined as follows:

1 $$P=\frac{dQ}{dt}\times \frac{1}{A{C}_{0}},$$

where *P* is the permeability coefficient, $dQ/dt$ is the diffusion rate of FITC-dextran, *A* is the surface area of the membrane, and *C*_0_ is the initial concentration of the FITC-dextran solution in the apical side of the well insert. The light transmittance of the four membranes was finally measured with a spectrophotometer (Epoch2, BioTek Instrument, USA) within the wavelength range of 360–700 nm.

### Cell culture and seeding

Human umbilical vein endothelial cells (HUVECs) were purchased from PromoCell (Germany). The HUVECs were cultured in endothelial cell growth medium 2 (EGM2, PromoCell, Germany) supplemented with 5 ng/ml EGF, 20 ng/ml R3 IGF-1, 0.5 ng/ml VEGF, 1 μg/ml ascorbic acid, 22.5 μg/ml heparin, and 0.2 μg/ml hydrocortisone, and the HUVECs were maintained in a humidified incubator at 37 °C with 5% CO_2_. The HUVECs were seeded on the four different membranes at a density of 1 × 10^5^ cells/cm^2^ for all the experiments, and the cells were used within passage six.

### Immunofluorescence imaging

After 5 days of cell culture, the samples were fixed in 4% paraformaldehyde solution (pH 7.4) for 15 min at room temperature and then permeabilized with 0.3% Triton™ X-100 (Sigma-Aldrich, USA) in PBS for 10 min at 4 °C. After three times of PBS washing, the samples were placed in a blocking buffer (5% normal goat serum and 1% bovine serum albumin in PBS-0.3% Triton-X100) for 1 h at room temperature. The samples were incubated with the diluted primary antibody solution containing mouse anti-ZO-1 (1:100; Thermo Fisher Scientific, USA) and rabbit anti-VE-cadherin (1:100; Cell Signaling Technology, USA), in a humidified chamber at 4 °C for overnight. The next day, the samples were washed with PBS 6 times, and secondary antibodies (goat anti-rabbit TRITC (1:400; Sigma-Aldrich, USA) and goat anti-mouse FITC (1:400; Sigma-Aldrich, USA) in the blocking buffer) were added. After 1 h of incubation in a dark chamber at 4 °C, the samples were washed 6 times with PBS, and the nuclei counterstaining was performed with DAPI (Sigma-Aldrich, USA) for 5 min. The samples were imaged with a confocal microscope (LSM 700, Carl Zeiss, Germany). The length and both angles of the major and minor axes of HUVECs were measured with ImageJ analysis software for the analysis of cell morphology in terms of aspect ratio and orientation angle.

### Transendothelial electrical resistance (TEER) measurement

TEER measurement is a noninvasive technique for indirectly evaluating the tight junction integrity of the cells through the measurement of the electrical resistance across a cellular layer. The evaluation of TEER value was performed by using a commercially available TEER measurement equipment (EVOM2, World Precision Instruments) with a chopstick electrode pair (STX3, World Precision Instruments, USA). The TEER values of Transwell membrane, Transwell-COL membrane, PCL-ANM, and Col-ANM were measured in triplicate. The TEER values were finally determined as follows:

2 $${\rm{TEER}}\,(\Omega \cdot {{\rm{cm}}}^{2})=({R}_{T}\,(\Omega )-{R}_{B}(\Omega ))\times A\,({{\rm{cm}}}^{2}),$$

where ${R}_{T}$ is the total resistance across the cellular monolayer on the membrane, ${R}_{B}$ is the blank resistance of the membrane only (without cells), and $A$ is surface area of the membrane (1.12 cm^2^ in this 12 well insert case). The triplicate average TEER values of the four different membranes were plotted each day by tracking 4 days of culture.

### Cellular permeability test

The permeability of the cellular layers formed on the four different membranes was evaluated when the TEER value reached the maximum value. The experimental method was the same as the membrane permeability test. The diffusion of 40 kDa FITC-dextran tracers across the membrane with cells was measured, and cellular permeability was calculated at day 4 of culture as follows^49^.

3 $$\frac{1}{{P}_{c}}=\frac{1}{{P}_{a}}-\frac{1}{{P}_{m}},$$

where ${P}_{a}$ is the apparent permeability coefficient (combined permeability of a cell layer and a membrane in this case), ${P}_{c}$ is the cellular permeability coefficient, and ${P}_{m}$ is the membrane permeability coefficient of the membrane only (without cells).

### Reactive oxygen species treatment

A defective barrier function in the endothelial monolayer tightly formed on the Col-ANM was examined by treating ROS over time. On day 5, a 500 mM H_2_O_2_ solution (Sigma-Aldrich) was added to the apical side of the Col-ANM well insert where an endothelial monolayer was formed. The ROS treatment was performed at room temperature for 1 h, and the changes in TEER values were measured every 15 min. The intercellular junction proteins of ZO-1 and VE-Cadherin were observed after 15 and 60 min of the ROS treatment for the visualization of endothelium dysfunction.

### Statistics

The results were expressed as means ± SD for the number of indicated determinations. Statistical significance of the differences was determined by one-way analysis of variance (ANOVA). A p value of < 0.05 was considered significant. Analyses were performed with GraphPad Prism software (GraphPad Software, USA)

## Supplementary information

Supplementary Material
