# Supplementary material for: A collagen gel-coated, aligned nanofiber membrane for enhanced endothelial barrier function
Source: Sci Rep. 2019 Oct 17;9:14915. doi: 10.1038/s41598-019-51560-8 (PMC6797789; doi:10.1038/s41598-019-51560-8)
Supplement: Supplementary file 1 — Supplementary Material [file 41598_2019_51560_MOESM1_ESM.docx]

A collagen gel-coated, aligned nanofiber membrane for enhanced endothelial barrier function

Dohui Kim^1, a)^, Seongsu Eom^1, a)^, Sang Min Park^1,2^, Hyeonjun Hong^1^ and Dong Sung Kim^1*^

^1^Department of Mechanical Engineering, Pohang University of Science and Technology (POSTECH), 77 Cheongam-ro Nam-gu, Pohang, Gyeongbuk 37673 South Korea

^2^Present Address: Department of Mechanical Engineering, Pusan National University, 2 Busandaehak-ro 63beon-gil, Busan, South Korea

*^a)^ These authors contributed equally to this work.*

*^*^Author to whom correspondence should be addressed; electronic mail: smkds@postech.ac.kr*

**1. SEM image of the Transwell**^®^ **membrane**

**
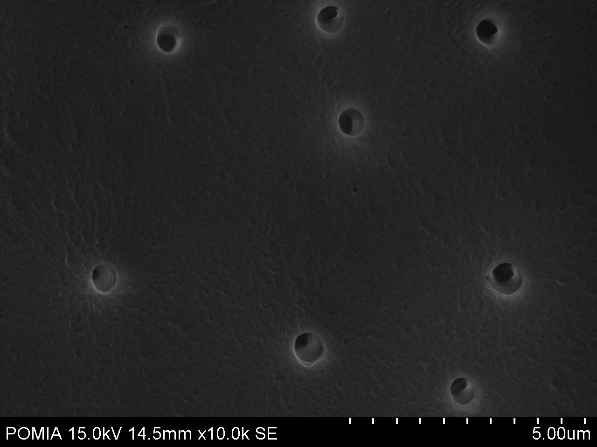
**

**Figure S1. SEM image of the Transwell membrane**

**2. Tuning the porosity and pore size of the PCL-ANM**

The tunability of the porosity and pore size of the PCL-ANM was demonstrated by fabricating PCL-ANMs with different electrospinning times of 15, 30, 45, and 60 min. We found that the longer the electrospinning time was, the smaller the porosity and pore size were obtained, based on the examination of SEM images (Fig. S2).


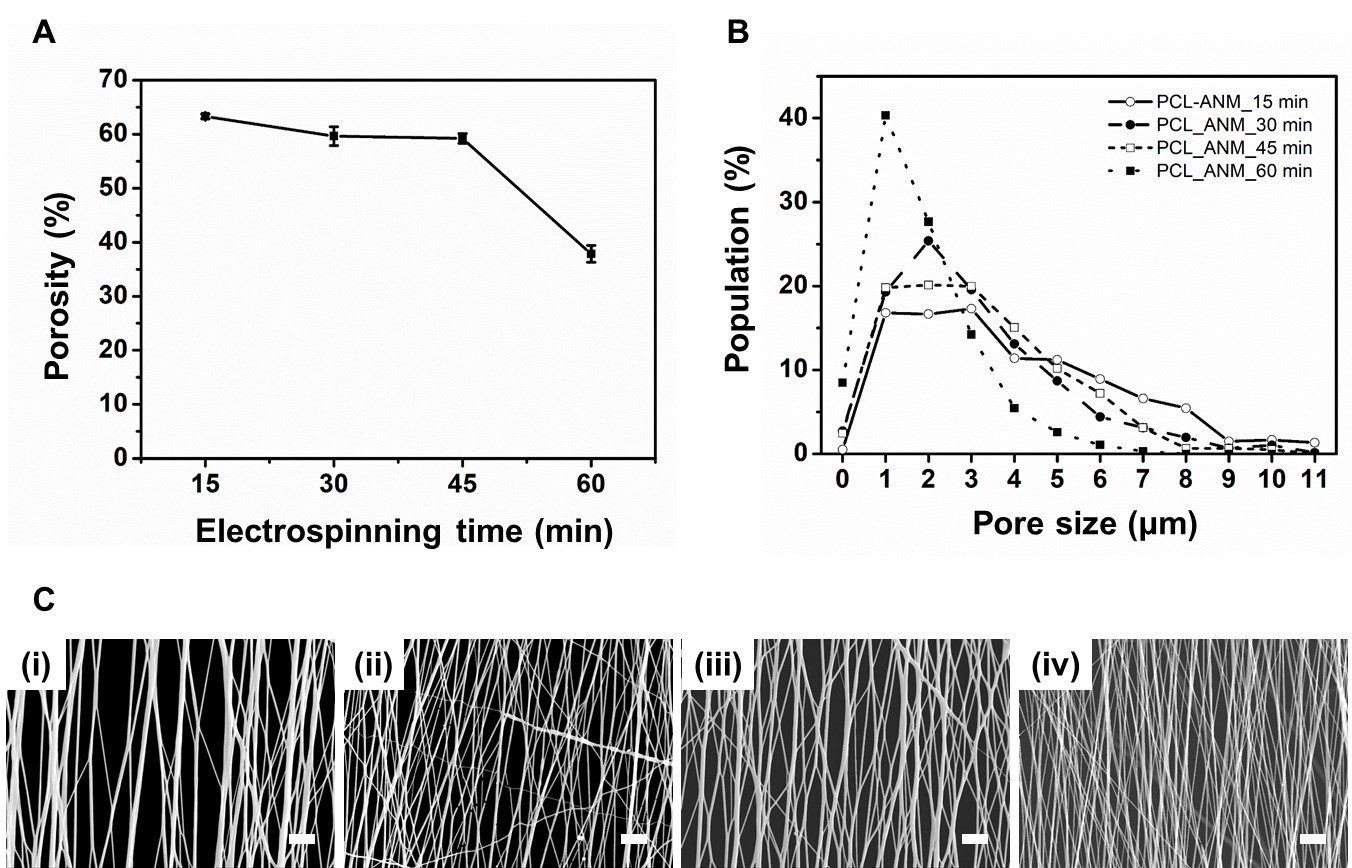


**Figure S2. Modulation of the porosity (A) and pore size distribution (B) of the PCL-ANM with respect to the electrospinning time. (C) SEM images of PCL-ANM with different electrospinning time of 15, 30, 45, and 60 min. Scale bars are10 μm.**

**3. Evaluation of the TEER value of the Col-ANM and Col-RNM**


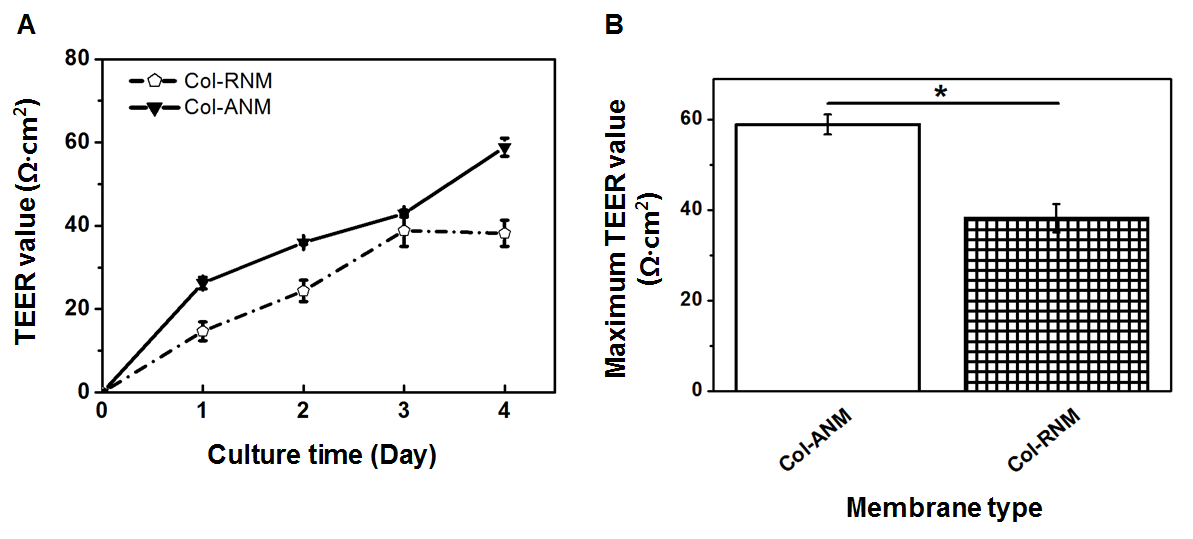


**Figure S3. Evaluation of the endothelial barrier integrity of HUVECs monolayer on Col-ANM compared with Col-RNM. (A) Change of TEER values of the HUVECs monolayer on the two different membranes during four days of culture. (B) Maximum TEER values of the HUVECs monolayers on the two different membranes.**
